# Oral health behaviors, perceptions, and educational needs among enlisted South Korean Army soldiers during the COVID-19 pandemic: A cross-sectional study

**DOI:** 10.1097/MD.0000000000050946

**Published:** 2026-10-02

**Authors:** Youmi Kim, Sojung Mun

**Affiliations:** a Department of Spinal Cord Injury Rehabilitation, National Rehabilitation Center, Ministry of Health and Welfare, Seoul, Republic of Korea; b Department of Dental Hygiene, College of Software and Digital Healthcare Convergence, Yonsei University, Wonju, Gangwon-do, Republic of Korea.

**Keywords:** COVID-19, oral health behaviors, oral health education, oral health perceptions, soldiers, South Korea

## Abstract

Preventive oral health care and education are essential for soldiers’ health and military readiness. This study investigated oral health behaviors, dental service utilization, oral health perceptions, and educational needs among enlisted South Korean Army soldiers during the coronavirus disease 2019 pandemic. A cross-sectional survey was conducted among 313 enlisted soldiers who visited military dental clinics in Gyeonggi-do, Republic of Korea, from March 24 to April 16, 2021. Descriptive statistics and Pearson’s χ^2^ tests were performed. Overall, 60.4% of participants did not use oral care products, mainly because they did not perceive a need for them (62.9%). Furthermore, 71.9% had not undergone routine dental examinations, 63.6% had not received dental treatment after enlistment, and 91.4% had no oral health education experience during military service. Video-based education was the preferred method (40.6%), and halitosis prevention and management was the most preferred topic (20.4%). Preferred educational methods did not differ significantly according to age, military rank, educational level, or smoking status (all *P* > .05). Military rank was significantly associated with perceived adequacy of oral care time (*P* < .001) and the place of dental treatment (*P* = .048). Routine dental examinations, dental treatment experience, and oral health education experience showed no significant differences according to participants’ general characteristics. Preventive oral health services and education were insufficient among enlisted South Korean Army soldiers. Tailored programs reflecting soldiers’ needs and preferred learning methods should be developed and evaluated.

## 1. Introduction

As society has developed, improving health and quality of life has become increasingly important.^[1]^ Oral health is an important determinant of quality of life because it affects communication, food intake, and overall well-being, and therefore requires appropriate management.^[2]^

The Ministry of Health and Welfare in South Korea conducts annual national oral health surveys to improve oral health policies and promote preventive oral healthcare.^[3]^ In addition, the Ministry of National Defense has implemented several initiatives to improve soldiers’ oral health, including the “Army Medical Program Improvement Plan” introduced in 2011 and mandatory dental examinations for soldiers who have served for >1 year.^[4,5]^

However, despite these policy changes, preventive oral health management remains insufficient, and routine oral examinations are not consistently provided to all soldiers.^[6,7]^ Consequently, South Korean soldiers continue to demonstrate poor oral health behaviors and limited access to routine dental care, resulting in a relatively high risk of oral diseases despite national efforts to improve oral health awareness.^[6,8,9]^

In Iraq, dental emergencies among military personnel cost approximately US$18 million per year, highlighting the importance of preventive oral healthcare and education.^[10]^ Similarly, the US military classifies soldiers into 4 dental readiness categories based on their oral health status and treatment needs.^[11]^ Soldiers requiring urgent dental treatment must complete treatment before deployment. Furthermore, the US Army provides regular oral health promotion messages on oral hygiene, tobacco cessation, and fluoride use^[12]^ and operates the TRICARE Dental Program, which supports continuous dental care for soldiers and their families. As a result, approximately 90% of US soldiers receive routine dental examinations during military service.^[8]^ Likewise, the Dutch military provides comprehensive medical and dental services through military healthcare facilities.^[13]^

These international examples emphasize the importance of preventive oral healthcare systems and structured oral health education for maintaining military readiness. In comparison, the Korean military still places greater emphasis on treatment than prevention. Oral health education is relatively inexpensive and can be effectively delivered through online or remote learning, regardless of location.^[14,15]^ Moreover, encouraging soldiers to perform appropriate self-care may contribute to reducing long-term dental treatment costs.

Previous studies on military health education have primarily focused on smoking cessation and nutrition.^[16,17]^ In contrast, evidence regarding oral health education and oral health behaviors among soldiers remains limited. This lack of evidence makes it difficult to develop effective oral health education programs tailored to military personnel.

During the coronavirus disease 2019 (COVID-19) pandemic, face-to-face education was restricted because of social distancing policies implemented by the Korean government and the Ministry of National Defense.^[18]^ Consequently, interest in remote and online educational approaches increased, creating a need for alternative methods of delivering oral health education to soldiers.^[19]^

Therefore, this study aimed to investigate the oral health behaviors, perceptions, and educational needs of enlisted South Korean Army soldiers during the COVID-19 pandemic. The findings of this cross-sectional study may provide evidence to support the development of tailored oral health education programs for military personnel.

## 2. Materials and methods

### 2.1. Study design and participants

This study employed a cross-sectional questionnaire-based survey to investigate the oral health behaviors, perceptions, and educational needs of enlisted South Korean Army soldiers during the COVID-19 pandemic.

Participants were recruited using a convenience sampling method from active-duty enlisted soldiers who visited the dental clinic at the Armed Forces Hospital, Gyeonggi-do, Republic of Korea, between March 24 and April 16, 2021. The study site was selected because it was readily accessible to the investigators and provided access to active-duty soldiers receiving routine dental care. The inclusion criteria were as follows: active-duty enlisted South Korean Army soldiers, soldiers who visited the military dental clinic during the study period, and those who voluntarily agreed to participate after receiving an explanation of the study purpose and procedures. The exclusion criteria were duplicate questionnaire responses, questionnaires with substantial missing data, incomplete questionnaires, and questionnaires containing logically inconsistent responses across related questionnaire items. These were defined as responses that were contradictory across related items.

The required sample size was calculated using G*Power version 3.1 (Heinrich Heine University Düsseldorf, Düsseldorf, Germany) with a significance level of 0.05, an effect size of 0.30, and a statistical power of 0.95. The minimum required sample size was 295 participants. Assuming a 10% dropout rate, 324 participants were recruited. A total of 324 questionnaires were returned. Of these, 11 were excluded because they met the predefined exclusion criteria, leaving 313 questionnaires for the final analysis.

### 2.2. Questionnaire

The questionnaire was developed based on the instrument previously reported by Park^[20]^ and was modified to reflect the objectives of the present study. The original questionnaire was revised by modifying the wording of several items to better reflect the military environment during the COVID-19 pandemic while maintaining the overall structure and content of the original instrument.

The final questionnaire consisted of 18 items, including 4 items on general characteristics, 10 items assessing oral health behaviors and perceptions, and 4 items assessing experiences with and demand for oral health education. Oral health perceptions were assessed using self-reported items on perceived adequacy of time available for oral care and subjective oral health status. These items were analyzed individually and were not combined into a composite score.

Prior to the main survey, the questionnaire was reviewed by the research team to evaluate the appropriateness, clarity, and relevance of each item for active-duty military personnel. A pilot survey involving 20 enlisted soldiers was subsequently conducted to assess the clarity, readability, comprehensibility, and feasibility of the questionnaire. Based on participants’ feedback, minor revisions were made to improve the wording and readability of several items before administration of the final questionnaire.

Because the questionnaire primarily consisted of independent factual and behavioral items rather than a multi-item psychometric scale, internal consistency reliability was not evaluated. The pilot survey confirmed that the questionnaire was understandable and appropriate for the target population.

### 2.3. Operational definitions

For the purpose of this study, oral care products were defined as oral hygiene devices used in addition to a toothbrush and toothpaste, including dental floss, interdental brushes, mouthwash, tongue cleaners, and other supplementary oral hygiene aids.

Routine dental examination was defined as a preventive dental examination performed in the absence of dental symptoms for the purpose of maintaining oral health rather than receiving treatment for existing oral diseases.

Oral health education experience was defined as having received any form of oral health education after military enlistment, regardless of the educational method or duration.

### 2.4. Ethical considerations

This study was approved by the Institutional Review Board of Wonju Severance Christian Hospital, Yonsei University (Approval No. CR320163). All participants received an explanation of the study objectives and procedures before participation, and written informed consent was obtained from all participants prior to completion of the questionnaire.

### 2.5. Statistical analysis

Statistical analyses were performed using IBM SPSS Statistics version 31.0 (IBM Corp., Armonk). Participants’ general characteristics, oral health behaviors, dental service utilization, and oral health education experience were summarized using descriptive statistics and presented as frequencies and percentages. Differences according to general characteristics were analyzed using Pearson’s χ^2^ test. Fisher’s exact test was used when the assumptions of the χ^2^ test were not satisfied because more than 20% of the expected cell counts were <5. All statistical tests were two-sided, and a *P* value of <.05 was considered statistically significant.

## 3. Results

### 3.1. General characteristics of the participants

A total of 313 military personnel participated in this study. The largest age group was 21 to 25 years (63.3%), followed by ≤20 years (32.5%) and 26 to 30 years (4.2%). Regarding military rank, corporals accounted for the largest proportion (46.1%), followed by private first class soldiers (31.9%), sergeants (17.2%), and privates (4.8%). Most participants were currently on leave from university (71.3%), while 51.5% were never smokers, 39.9% were current smokers, and 8.6% were former smokers (Table 1).

**Table 1:** General characteristics (N = 313).

Characteristic	n (%)
Age	
≤20 yr	102 (32.5)
21–25 yr	198 (63.3)
26–30 yr	13 (4.2)
Rank	
Private	15 (4.8)
Private first class	100 (31.9)
Corporal	144 (46.1)
Sergeant	54 (17.2)
Education level	
High school graduate or lower	60 (19.2)
Currently on leave from university	223 (71.3)
University graduate or above	30 (9.5)
Smoking	
Never smoker	161 (51.5)
Former smoker	27 (8.6)
Current smoker	125 (39.9)

### 3.2. Oral health behaviors according to general characteristics

Table 2 shows the comparison of oral health behaviors according to general characteristics. Overall, more than 90% of participants reported having sufficient time for oral care across all age groups, educational levels, and smoking status categories. Most participants brushed their teeth 2 times per day or ≥3 times per day, and the largest proportion reported brushing for 2 to <3 minutes per session. Among the general characteristics examined, only military rank was significantly associated with the perceived adequacy of time for oral care (χ^2^ = 88.39, *P* < .001). No significant differences were found in toothbrushing frequency or duration according to age, military rank, educational level, or smoking status (*P* > .05).

**Table 2:** Comparison of oral health behaviors according to general characteristics (N = 313).

Characteristic	Category	Time for oral care			Frequency of toothbrushing				Toothbrushing time					Use of oral care products			Subjective oral health			
		Enough	Not enough	χ^2^ (*P*)	Once or less	Twice	Thrice or more	χ^2^ (*P*)	<1 min	1–<2 min	2–<3 min	≥3 min	χ^2^ (*P*)	Yes	No	χ^2^ (*P*)	Healthy	Normal	Unhealthy	χ^2^ (*P*)
Age	≤20 yr	92 (95.8)	4 (4.2)	3.43 (.181)	7 (6.9)	48 (47.1)	47 (46.1)	2.58 (.630)	4 (3.9)	31 (30.4)	49 (48.0)	18 (17.6)	1.86 (.932)	37 (38.5%)	59 (61.5%)	0.08 (.961)	21 (20.6)	56 (54.9)	25 (24.5)	0.23 (.994)
	21–25 yr	187 (91.7)	17 (8.3)		11 (5.6)	92 (46.5)	95 (48.0)		7 (3.5)	60 (30.3)	96 (48.5)	35 (17.7)		82 (40.2%)	122 (59.8%)		39 (19.7)	109 (55.1)	50 (25.3)	
	26–30 yr	12 (92.3)	1 (7.7)		1 (7.7)	4 (30.8)	8 (61.5)		0 (0.0)	4 (30.8)	6 (46.2)	3 (23.1)		5 (38.5%)	8 (61.5)		3 (23.1)	7 (53.8)	3 (23.1)	
Rank	Private	13 (39.4)	20 (60.6)	88.39 (<.001)***	2 (6.1)	16 (48.5)	15 (45.5)	1.24 (.975)	2 (6.1)	10 (30.3)	16 (48.5)	5 (15.2)	2.87 (.825)	12 (36.4)	21 (63.6)	0.42 (.936)	6 (18.2)	18 (54.5)	9 (27.3)	0.92 (.988)
	Private first class	89 (92.7)	7 (7.3)		6 (6.3)	46 (47.9)	44 (45.8)		3 (3.1)	30 (31.3)	46 (47.9)	17 (17.7)		37 (38.5)	59 (61.5)		20 (20.8)	53 (55.2)	23 (24.0)	
	Corporal	129 (96.3)	5 (3.7)		8 (6.0)	60 (44.8)	66 (49.3)		5 (3.7)	40 (29.9)	66 (49.3)	23 (17.2)		56 (41.8)	78 (58.2)		28 (20.9)	73 (54.5)	33 (24.6)	
	Sergeant	46 (92.0)	4 (8.0)		3 (6.0)	22 (44.0)	25 (50.0)		1 (2.0)	15 (30.0)	23 (46.0)	11 (22.0)		19 (38.0)	31 (62.0)		9 (18.0)	28 (56.0)	13 (26.0)	
Education level	High school graduate or lower	54 (90.0)	6 (10.0)	2.28 (.320)	5 (8.3)	28 (46.7)	27 (45.0)	1.49 (.829)	3 (5.0)	19 (31.7)	28 (46.7)	10 (16.7)	1.63 (.950)	22 (36.7)	38 (63.3)	0.33 (.849)	11 (18.3)	33 (55.0)	16 (26.7)	0.41 (.981)
	Currently on leave from university	211 (94.6)	12 (5.4)		13 (5.8)	102 (45.7)	108 (48.4)		7 (3.1)	68 (30.5)	108 (48.4)	40 (17.9)		90 (40.4)	133 (59.6)		46 (20.6)	123 (55.2)	54 (24.2)	
	University graduate or above	29 (96.7)	1 (3.3)		1 (3.3)	14 (46.7)	15 (50.0)		1 (3.3)	8 (26.7)	15 (50.0)	6 (20.0)		12 (40.0)	18 (60.0)		6 (20.0)	16 (53.3)	8 (26.7)	
Smoking	Never smoker	152 (94.4)	9 (5.6)	0.33 (.847)	8 (5.0)	75 (46.6)	78 (48.4)	0.78 (.941)	5 (3.1)	49 (30.4)	79 (49.1)	28 (17.4)	0.74 (.993)	65 (40.4)	96 (59.6)	0.12 (.943)	34 (21.1)	87 (54.0)	40 (24.8)	0.36 (.986)
	Former smoker	25 (92.6)	2 (7.4)		2 (7.4)	12 (44.4)	13 (48.1)		1 (3.7)	8 (29.6)	13 (48.1)	5 (18.5)		10 (37.0)	17 (63.0)		5 (18.5)	15 (55.6)	7 (25.9)	
	Current smoker	117 (93.6)	8 (6.4)		9 (7.2)	57 (45.6)	59 (47.2)		5 (4.0)	38 (30.4)	59 (47.2)	23 (18.4)		49 (39.2)	76 (60.8)		24 (19.2)	70 (56.0)	31 (24.8)	

*** *P* < .001.

### 3.3. Dental service utilization according to general characteristics

No significant differences were found in regular oral examinations or dental treatment experience according to age, military rank, educational level, or smoking status (*P* > .05). However, the place of dental treatment was significantly associated with military rank (χ^2^ = 7.91, *P* = .048). The proportion of participants receiving dental treatment at military hospitals varied across military rank categories. No significant associations with the place of dental treatment were observed according to age, educational level, or smoking status (Table 3).

**Table 3:** Comparison of dental service utilization according to general characteristics (N = 313).

Characteristic	Category	Routine dental examinations			Dental treatment			Place of dental treatment		
		Yes	No	χ^2^ (*P*)	Yes	No	χ^2^ (*P*)	Army hospital	Civilian clinic	χ^2^ (*P*)
Age	≤20 yr	26 (25.5)	76 (74.5)	0.51 (.774)	31 (30.4)	71 (69.6)	5.74 (.057)	78 (76.5)	24 (23.5)	3.96 (.138)
	21–25 yr	58 (29.3)	140 (70.7)		72 (36.4)	126 (63.6)		143 (72.2)	55 (27.8)	
	26–30 yr	4 (30.8)	9 (69.2)		8 (61.5)	5 (38.5)		7 (53.8)	6 (46.2)	
Rank	Private	7 (21.2)	26 (78.8)	3.82 (.282)	9 (27.3)	24 (72.7)	4.89 (.180)	29 (87.9)	4 (12.1)	7.91 (.048)*
	Private first class	24 (25.0)	72 (75.0)		30 (31.3)	66 (68.7)		72 (75.0)	24 (25.0)	
	Corporal	43 (32.1)	91 (67.9)		52 (38.8)	82 (61.2)		90 (67.2)	44 (32.8)	
	Sergeant	14 (28.0)	36 (72.0)		20 (40.0)	30 (60.0)		37 (74.0)	13 (26.0)	
Education level	High school graduate or lower	15 (25.0)	45 (75.0)	0.42 (.812)	18 (30.0)	42 (70.0)	0.89 (.641)	47 (78.3)	13 (21.7)	1.52 (.468)
	Currently on leave from university	64 (28.7)	159 (71.3)		82 (36.8)	141 (63.2)		161 (72.2)	62 (27.8)	
	University graduate or above	9 (30.0)	21 (70.0)		11 (36.7)	19 (63.3)		20 (66.7)	10 (33.3)	
Smoking	Never smoker	50 (31.1)	111 (68.9)	4.67 (.097)	63 (39.1)	98 (60.9)	2.84 (.242)	120 (74.5)	41 (25.5)	0.58 (.749)
	Former smoker	9 (33.3)	18 (66.7)		10 (37.0)	17 (63.0)		20 (74.1)	7 (25.9)	
	Current smoker	29 (23.2)	96 (76.8)		38 (30.4)	87 (69.6)		88 (70.4)	37 (29.6)	

* *P* < .05.

### 3.4. Oral health education experience and educational needs according to general characteristics

More than 90% of participants had not received oral health education during military service, while most participants expressed a need for oral health education. No significant differences in oral health education experience or educational needs were observed according to general characteristics (*P* > .05). Video-based education was the most preferred educational method, with no significant differences in preferred educational methods according to age, military rank, educational level, or smoking status (all *P* > .05, Table 4).

**Table 4:** Comparison of oral health education experience and educational needs according to general characteristics (N = 313).

Characteristic	Category	Oral health education experience			Need for oral health education			Preferred methods of oral health education			
		Yes	No	χ^2^ (*P*)	Yes	No	χ^2^ (*P*)	Video	Lecture	Printed materials	χ^2^ (*P*)
Age	≤20 yr	8 (7.8)	94 (92.2)	0.88 (.645)	90 (88.2)	12 (11.8)	0.41 (.814)	40 (39.2)	31 (30.4)	31 (30.4)	0.71 (.950)
	21–25 yr	17 (8.6)	181 (91.4)		177 (89.4)	21 (10.6)		81 (40.9)	60 (30.3)	57 (28.8)	
	26–30 yr	2 (15.4)	11 (84.6)		11 (84.6)	2 (15.4)		6 (46.2)	4 (30.8)	3 (23.1)	
Rank	Private	2 (6.1)	31 (93.9)	0.39 (.942)	27 (81.8)	6 (18.2)	4.82 (.186)	15 (45.5)	10 (30.3)	8 (24.2)	1.94 (.925)
	Private first class	8 (8.3)	88 (91.7)		87 (90.6)	9 (9.4)		39 (40.6)	29 (30.2)	28 (29.2)	
	Corporal	13 (9.7)	121 (90.3)		122 (91.0)	12 (9.0)		51 (38.1)	42 (31.3)	41 (30.6)	
	Sergeant	4 (8.0)	46 (92.0)		42 (84.0)	8 (16.0)		22 (44.0)	14 (28.0)	14 (28.0)	
Education level	High school graduate or lower	4 (6.7)	56 (93.3)	0.57 (.754)	51 (85.0)	9 (15.0)	1.08 (.582)	22 (36.7)	20 (33.3)	18 (30.0)	0.64 (.958)
	Currently on leave from university	20 (9.0)	203 (91.0)		200 (89.7)	23 (10.3)		93 (41.7)	66 (29.6)	64 (28.7)	
	University graduate or above	3 (10.0)	27 (90.0)		27 (90.0)	3 (10.0)		12 (40.0)	9 (30.0)	9 (30.0)	
Smoking	Never smoker	16 (9.9)	145 (90.1)	0.74 (.691)	147 (91.3)	14 (8.7)	2.36 (.307)	68 (42.2)	47 (29.2)	46 (28.6)	0.84 (.991)
	Former smoker	2 (7.4)	25 (92.6)		23 (85.2)	4 (14.8)		10 (37.0)	8 (29.6)	9 (33.3)	
	Current smoker	9 (7.2)	116 (92.8)		108 (86.4)	17 (13.6)		49 (39.2)	40 (32.0)	36 (28.8)	

## 4. Discussion

We assessed the oral health behaviors, dental service utilization, and oral health education experience of enlisted South Korean Army soldiers during the COVID-19 pandemic to identify current gaps in oral health promotion. The findings provide evidence for developing effective preventive oral health strategies and oral health education programs tailored to military personnel. This study analyzed oral health behaviors, oral health perceptions, utilization of dental institutions, dental treatment experience, and preferred methods and content of oral health education.

We observed that 60.4% of the soldiers did not use oral care products other than toothbrushes and toothpaste. Consistent with our findings, Škec et al^[21]^ reported that 88.4% of soldiers did not use additional oral care products. The main reasons for not using oral care products were “not knowing which products to use” and “not feeling the need to use them.” Similar findings have been reported among Korean university students by Seong and Moon,^[22]^ Kim,^[23]^ and Lee and Lee,^[24]^ indicating that awareness of oral care products remains low among young adults. These findings suggest insufficient exposure to preventive oral health education.^[9]^ Soldiers generally have limited access to dental education compared with the general population.^[6]^ Therefore, improving awareness of oral care products and providing continuous preventive oral health education may contribute to promoting better oral hygiene practices among military personnel.

Approximately 71.9% of participants did not receive routine dental examinations. Similar findings have been reported by Hong,^[25]^ Jung and Jung,^[6]^ and Kim et al,^[26]^ who found that 73.9%, 95.0%, and 82.6% of soldiers, respectively, did not undergo routine dental examinations. Routine dental examinations facilitate early detection and treatment of oral diseases and contribute to maintaining long-term oral health.^[27]^ Although most participants recognized the importance of routine dental examinations, the utilization rate remained low. Furthermore, no significant differences in routine dental examinations were observed according to age, military rank, educational level, or smoking status. This finding suggests that the low utilization of preventive dental services is a widespread issue across military personnel rather than being limited to specific demographic groups. Therefore, military-wide strategies should be implemented to encourage routine dental examinations.

Unlike our findings, only 19.5% and 20.0% of soldiers in Croatia and the United States reportedly did not receive routine dental examinations.^[19]^ In Croatia, routine dental examinations are considered an important indicator of oral health management.^[19]^ In the United States, the Directorate of Health Promotion and Wellness integrates preventive dental services into military health promotion programs.^[12]^ The higher participation in preventive dental examinations observed in these countries may reflect well-established preventive oral health systems. Therefore, the Korean military should strengthen preventive oral health policies and establish systematic oral health promotion programs.

Although military regulations require soldiers to receive dental treatment initially at military hospitals, only 60.4% of participants reported receiving treatment at military hospitals. Yoo^[28]^ similarly reported that 52.7% of soldiers received dental treatment at military hospitals. Previous studies have suggested that limited access to medical facilities and the geographical location of military bases contribute to this pattern.^[29]^ The most common reason for visiting civilian dental clinics was a lack of trust in military hospitals, which is consistent with previous findings reported by Lee et al.^[5]^ According to data from the Ministry of National Defense,^[30]^ the number of military dentists remains insufficient relative to the number of active-duty soldiers, potentially limiting the quality and accessibility of dental services.

Interestingly, the place of dental treatment was significantly associated with military rank (*P* = .048), whereas no significant differences were observed according to age, educational level, or smoking status. Although the distribution of dental treatment locations varied across military rank categories, specific differences between individual rank groups were not evaluated. Further research is needed to clarify the factors underlying this association.

Another noteworthy finding was that perceived adequacy of time available for oral care was significantly associated with military rank. Lower-ranked soldiers, particularly privates, were less likely to report having sufficient time for oral care than soldiers of higher ranks. This difference may be associated with differences in workload, daily schedules, or opportunities for personal hygiene. Organizational efforts to ensure adequate time for oral hygiene, particularly among junior soldiers, may help improve oral health behaviors in the military.

Approximately 91.4% of participants reported that they had never received oral health education after enlistment. Similar findings have been reported by Park,^[20]^ Jung and Jung,^[6]^ and Yoo and Kim,^[28]^ indicating that the majority of Korean soldiers have consistently lacked opportunities to receive oral health education over the past 2 decades. These findings suggest persistent deficiencies in preventive oral health services and oral health promotion within the military.^[31]^ Furthermore, no significant differences in oral health education experience or educational needs were observed according to age, military rank, educational level, or smoking status. This indicates that the lack of oral health education affects military personnel regardless of demographic characteristics, highlighting the need for comprehensive educational programs throughout the military.

Approximately 40.6% of participants preferred video-based education, while the prevention and management of halitosis was the most preferred educational topic. Similar preferences for video-based education have been reported by Yoo and Kim^[28]^ and Park.^[31]^ Considering the expansion of digital learning environments during the COVID-19 pandemic, video-based education may provide an efficient and scalable approach for delivering oral health education to military personnel. Video materials are easily accessible, can be repeatedly viewed, and require relatively few educational resources. However, combining video education with practical training may further improve educational effectiveness.^[32–34]^ Increased mask use during the COVID-19 pandemic may also have heightened awareness of halitosis.^[35]^ Because most causes of halitosis originate in the oral cavity,^[36]^ education focusing on halitosis prevention may motivate soldiers to adopt healthier oral hygiene behaviors.

Several limitations should be considered when interpreting these findings. First, participants were recruited using a convenience sampling method from a single military dental clinic in one region, which may have introduced selection bias and limited the generalizability of the findings to all South Korean military personnel. Second, because the data were collected using self-reported questionnaires, recall bias and social desirability bias may have influenced participants’ responses. Third, the cross-sectional study design precludes the establishment of causal relationships between general characteristics and oral health behaviors. Fourth, although significant associations with military rank were identified for several variables, the unequal distribution of participants across military ranks may have influenced the statistical power and interpretation of subgroup analyses. Finally, the data were collected during a specific period of the COVID-19 pandemic in 2021, and oral health behaviors and educational needs may have changed over time. Therefore, future studies should recruit more representative samples from multiple military regions and branches and evaluate the effectiveness of tailored oral health education programs using prospective or interventional study designs.

Despite these limitations, this study provides meaningful evidence regarding oral health behaviors, dental service utilization, and oral health education needs among active-duty Korean soldiers during the COVID-19 pandemic. The findings demonstrate that preventive oral health services remain insufficient regardless of demographic characteristics, while military rank was associated with certain oral health perceptions and dental service utilization. These results provide important baseline evidence for developing systematic oral health promotion policies and tailored educational programs within the Korean military. Future research should develop and evaluate evidence-based oral health education programs that reflect soldiers’ preferred educational methods and content while promoting preventive oral health behaviors.

## Author contributions

**Conceptualization:** Youmi Kim, Sojung Mun.

**Formal analysis:** Youmi Kim.

**Methodology:** Youmi Kim, Sojung Mun.

**Software:** Youmi Kim.

**Supervision:** Sojung Mun.

**Writing – original draft:** Youmi Kim, Sojung Mun.

**Writing – review & editing:** Youmi Kim.
